# The Dynamic Life of Virus Capsids

**DOI:** 10.3390/v12060618

**Published:** 2020-06-05

**Authors:** Michael B. Sherman, Hong Q. Smith, Thomas J. Smith

**Affiliations:** Department of Biochemistry and Molecular Biology, University of Texas Medical Branch at Galveston, 301 University Boulevard, Route 0645, Galveston, TX 77555, USA; mbsherma@UTMB.EDU (M.B.S.); hqsmith@utmb.edu (H.Q.S.)

**Keywords:** rhinovirus, norovirus, antibodies, flexibility

## Abstract

Protein-shelled viruses have been thought as “tin cans” that merely carry the genomic cargo from cell to cell. However, through the years, it has become clear that viruses such as rhinoviruses and caliciviruses are active and dynamic structures waiting for the right environmental cues to deliver their genomic payload to the host cell. In the case of human rhinoviruses, the capsid has empty cavities that decrease the energy required to cause conformational changes, resulting in the capsids “breathing”, waiting for the moment when the receptor binds for it to release its genome. Most strikingly, the buried N-termini of VP1 and VP4 are transiently exposed during this process. A more recent example of a “living” protein capsid is mouse norovirus (MNV). This family of viruses have a large protruding (P) domain that is loosely attached to the shell via a single-polypeptide tether. Small molecules found in the gut, such as bile salts, cause the P domains to rotate and collapse onto the shell surface. Concomitantly, bile alters the conformation of the P domain itself from one that binds antibodies to one that recognizes receptors. In this way, MNV appears to use capsid flexibility to present one face to the immune system and a completely different one to attack the host tissue. Therefore, it appears that even protein-shelled viruses have developed an impressive array of tricks to dodge our immune system and efficiently attack the host.

## 1. Introduction

Non-enveloped viruses have been thought to have protein shells that simply move the viral genome from cell to cell. The following review will discuss human rhinovirus (HRV) and mouse norovirus (MNV) which intricately respond to environmental cues. With HRV14 (formal designation now HRV B14), the capsids undergo a “breathing” process, where the buried N-termini are transiently extruded [1]. This breathing is essential for the infection and is utilized by the receptor to initiate the uncoating process. While such a mobile capsid might be leveraged by antibodies for neutralization, that appears to not be the case. With MNV, the protruding (P) domain is only loosely tethered to the shell. In the presence of gut compounds such as bile salts, the P domain rotates down onto the shell and the conformation of the epitope is drastically changed [2]. It is likely that both changes optimize receptor/P domain interactions while affecting antibody recognition.

## 2. Rhinoviruses

**Figure 1:**
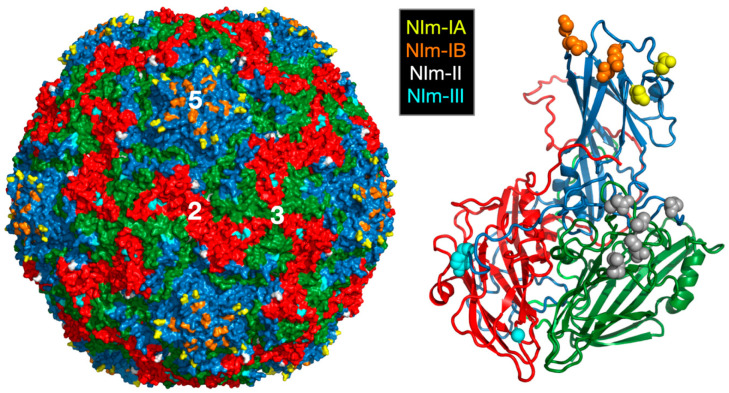
Structure of HRV14 and location of the NIm sites. Shown on the left is the surface of a pseudo T = 3 icosahedral capsid. VP1, VP2, and VP3 are shown in blue, green, and red, respectively. The locations of the escape mutation clusters are highlighted as noted. The right figure shows one icosahedral asymmetric unit using the same color scheme.

Picornaviruses are among the largest of animal virus families and include polio-, rhino-, foot-and-mouth disease, Coxsackie, and hepatitis A viruses. The rhinoviruses—of which, there are more than 100 serotypes—are major causative agents of the common cold in humans [3]. The virus is non-enveloped and has a ~300Å diameter protein shell that encapsidates a single-stranded, plus-sense, RNA genome of ~7200 bases. The human rhinovirus 14 (HRV14) capsid has pseudo T = 3 (P = 3) icosahedral symmetry and consists of 60 copies each of four viral proteins—VP1, VP2, VP3, and VP4 (Figure 1). VP1–3 each have an eight-stranded antiparallel β-barrel motif and comprise most of the capsid structure. VP4 is smaller, has an extended structure, and lies at the RNA–capsid interface [4]. A ~20Å deep canyon lies approximately at the junction of VP1 (forming the “north” rim) with VP2 and VP3 (forming the “south” rim), and surrounds each of the twelve icosahedral 5-fold vertices. The canyon regions of the major receptor group rhinoviruses, were shown to contain the binding site of the cellular receptor, intercellular adhesion molecule 1 (ICAM-1) [5,6,7]. Four major neutralizing immunogenic (NIm) sites—NIm-IA, NIm-IB, NIm-II, and NIm-III—were identified from neutralization escape mutants using monoclonal antibodies [8,9], and then mapped to four regions on the viral surface (Figure 1) [4].

### 2.1. The Canyon and Capsid “Breathing”

#### 2.1.1. Mechanism of Antiviral Compounds

A hydrophobic cavity lies directly beneath the canyon floor into which some non-polar antiviral compounds (WIN) bind [10]. Upon binding, these compounds greatly stabilize the capsid against thermal and pH denaturation [11] while causing only small changes in the canyon floor upon binding [10]. These hydrophobic compounds create an opening to the binding pocket by displacing M221 of VP1 [10,12,13]. Since the drug-induced conformational changes are limited to residues immediately surrounding the drug, it is likely that the major effect of the drug is stabilization of the capsid due to global Gibbs free energy effects of these compounds binding in a hydrophobic pocket rather than inducing gross changes in the capsid.

**Figure 2:**
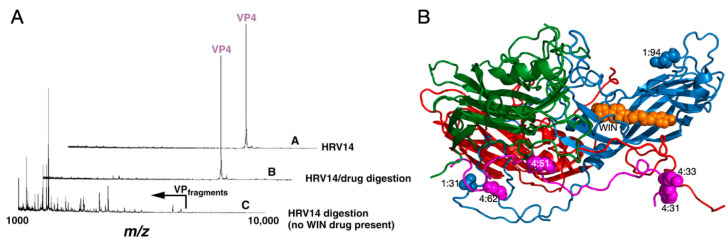
Use of mass spectrometry to demonstrate HRV14 breathing. (**A**) MALDI analysis of limited trypsinolysis of HRV14 in the presence and absence of WIN compounds. While HRV14 is extremely sensitive to trypsin cleavage at room temperature, the presence of WIN compounds blocks all cleavage for >18 h. (**B**) Ribbon diagram of an icosahedra asymmetric unit with VP1, VP2, VP3, and VP4 colored blue, green, red, and mauve, respectively. The locations of trypsin cleavage that occur within the first five minutes of exposure to trypsin are noted by spherical models. Note that there are cleavage sites at the extreme N-termini of both VP1 and VP4 not visible in the crystal structure.

Analysis of HRV14 trypsin digestion products using mass spectrometry provided evidence that the drug-binding cavity plays a role in capsid dynamics [1]. HRV14 was treated with immobilized trypsin for varying periods of times and the resulting fragments were analyzed to determine the sites of proteolytic cleavage (Figure 2). Unexpectedly, the sites most sensitive to trypsin cleavage are at the N-termini of VP1 and VP4 that are buried at the RNA–capsid interface. Within five minutes, there was cleavage at residues 13, 31, and 94 in VP1 and residues 9, 31, 33, 51, 62, and 94 in VP4. Since the virus concentration was in extreme excess, these represent single hit kinetics and are, therefore, independent and non-sequential events. This suggested that, even at room temperature, there is transient extrusion of these buried termini, or “breathing”.

It is possible that the digestion seen in these experiments was due to a mixture of intact and damaged HRV14 particles. To control for this, WIN compounds were added to the digestion mix since they were known to stabilize the HRV capsid. WIN compounds completely blocked cleavage at all sites but the drug did not affect the intrinsic activity of trypsin itself (Figure 2). This stabilization is rather extreme in that HRV14 alone is cleaved by trypsin within five minutes, whereas the presence of WIN compounds delays cleavage for more than 18 h. Interestingly, not only did the WIN compounds block cleavage of the buried N-termini but also at the exposed NIm-IA site (residue 94 of VP1) that is more than 30Å away from the pocket. This suggests that there is a great deal of motion throughout the HRV capsid and that, when these antiviral compounds bind to the base of the canyon, infectivity is blocked by abrogating this flexibility.

Further evidence of the stabilizing effects of the WIN compounds on HRV14 came from experiments where the virus was subjected to high hydrostatic pressure, low temperature, and urea in the absence and presence of an antiviral drug [14]. Capsid dissociation and changes in the protein conformation were monitored by fluorescence spectroscopy, light scattering, circular dichroism, gel filtration chromatography, mass spectrometry and infectivity assays. The data show that high pressure induces the dissociation of HRV14 and that this process is inhibited by WIN 52084. MALDI-TOF mass spectrometry experiments demonstrate that VP4, the most internal viral protein, is released from the capsid by pressure treatment. This release of VP4 is concomitant with loss of infectivity. These results are consistent with the model, whereby WIN drugs block capsid dynamics necessary for cell attachment.

WIN compounds have also been shown to affect the receptor-virus interactions of the major but not the minor serotypes of HRV [15]. On this basis, it was hypothesized that the small conformational changes in the canyon floor induced by drug binding were responsible for abrogation of cell attachment [14]. This hypothesis was attractive because of its conceptual simplicity. However, the minor group of HRV serotypes, that are also affected by the WIN drugs, utilize the LDL receptor [16,17] that binds to the top of the canyon [18]. It seems more likely that the mode of action of the compounds is stabilization of the capsid of all target viruses. The effects of the WIN drugs on ICAM binding is most likely unrelated to the minor conformational changes associated with drug binding. It is known that ICAM binding to the major HRV serotype group induces uncoating [19]. It is likely that, as ICAM interacts with the canyon region, large conformational changes occur to optimize receptor binding and this leads to uncoating. The binding of the WIN compounds stabilizes the capsid that likely not only prevents uncoating but also the receptor/HRV binding cooperativity.

#### 2.1.2. Antiviral Binding Cavity and “Pocket Factors”

**Figure 3:**
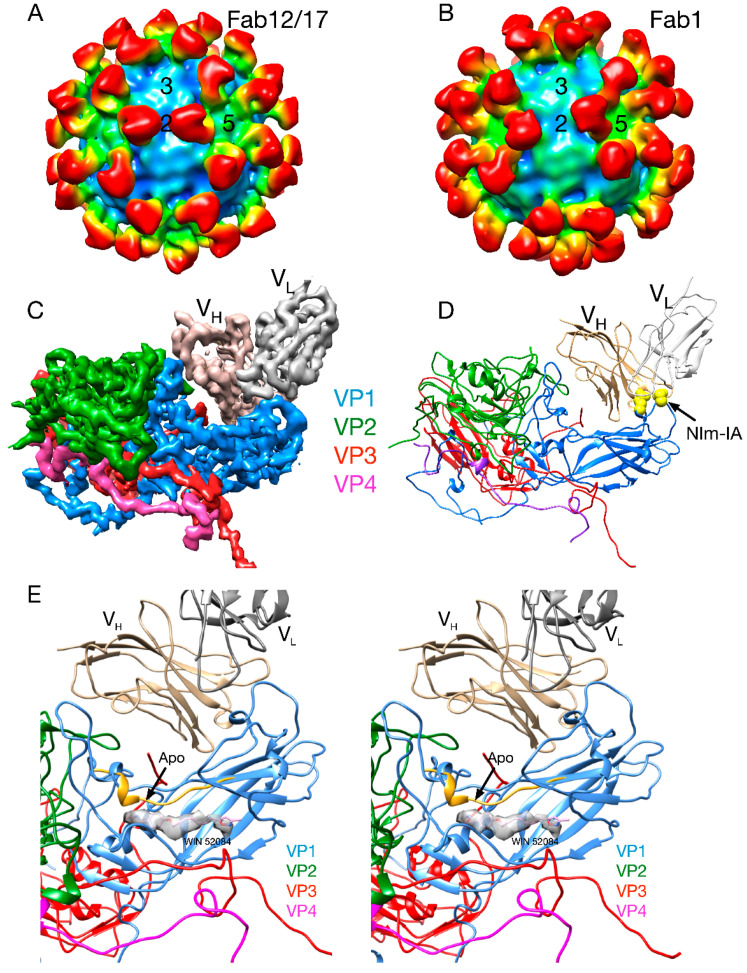
Early cryo-EM image reconstructions of Fab17 (**A**) and Fab1 (**B**) bound to HRV14 at ~20Å resolution. (**C**) Electron density of the 3.5Å crystal structure of the Fab17/HRV14 complex [21]. (**D**) The ribbon structure of the Fab17/HRV complex. The two Nim-IA escape mutants are highlighted in yellow. (**E**) Stereo diagram of the electron density of “pocket factor” (grey) in the Fab17/HRV14 crystal structure that came from the PEG400 used for freezing the crystals. For reference, the WIN 52084 drug [10,12] is shown and also moves the protein strand above the density by more than 3.5Å away from the apo conformation (yellow).

It was suggested that certain host cell molecules, “pocket factors,” bind to the WIN drug-binding cavity beneath the canyon floor and provide transient stability to some of the picornaviruses [20]. There were small islands of electron density in the WIN drug-binding cavity of a number of picornaviruses (but not HRV14) that were called “pocket factors.” It was proposed that host cells produce various pocket factors that stabilize virions in the extracellular milieu and these factors compete with receptor for binding to the virion. To explain how the drugs and receptor could regulate uncoating in the presence of pocket factors, it was proposed that WIN compounds and receptor bind better than the pocket factor. However, the t1/2 of WIN compounds disassociating from HRV14 is on the order of 30–90 min [11] and, therefore, it is unclear how these compounds can remain bound to virions during lengthy purification procedures. Most importantly, the structure of the Fab17/HRV14 complex [21] showed clear evidence for a bound pocket factor that was absent in the HRV14 structure alone [4]. This density must have come from the PEG 400 that was used as a cryo-solvent to freeze the crystals (Figure 3E). This suggests that the previously observed pocket factors may not have been host derived but rather from small-molecular-weight solutes in crystallization liquors or from additives used in the purification protocol. While these results suggest that the small molecules observed in the HRV crystal structures may not be from the cell, it was still possible that pocket factors exist in vivo.

#### 2.1.3. Antiviral Resistant Mutants

To test whether putative “pocket factors” might play a role in the viral life cycle, HRV14 was mutated in (V1188M, C1199W, and V1188M/C1199W) and around (S1223G) the drug-binding pocket [22]. Infectivity, limited proteolysis, and matrix-assisted laser desorption ionization analyses indicate that filling the drug-binding pocket with bulky side chains is not deleterious to viral replication but does stabilize the capsid. In contrast, studies with the S1223G mutant suggest that this mutation at least partially overcomes WIN drug-mediated inhibition of cell attachment and capsid breathing by lending some flexibility to the region. Finally, HRV16, which is inherently more stable than HRV14, was found to “breathe” only at 37 °C and did not tolerate stabilizing mutations in the drug-binding cavity. These results suggest that it is the drug-binding cavity itself and not the putative pocket factor(s) that is crucial for the capsid dynamics, which is, in turn, necessary for infection.

This simplified model is supported by studies demonstrating that drug-resistant mutations can arise distal to the drug-binding region. While some of the mutations that confer resistance in HRV14 make direct contact with the bound drug, many of the other residues point away from the bound WIN compounds and most lie on strands that remain relatively unchanged by drug binding. Therefore, these latter mutations may facilitate the canyon-associated conformational changes in the presence of drug rather than simply affecting receptor or drug binding.

HRV16 is a better example of distal, drug-resistant mutations. Resistant mutations V1210A, M1103T, and T1208A lie midway up the south canyon wall. These mutations destabilize virions, and hence, virions can only assemble in the presence of drug. This is an important finding in that it demonstrates that crucial residues are not only confined to the very bottom of the canyon and that the canyon region is very sensitive to mutations. Similarly, drug-resistant mutations are found in three regions of poliovirus: the drug-binding pocket itself, the north wall of the canyon (~17 Å away from the bound drug), and at the viral capsid inner surface. A number of these latter mutations are clustered around the VP4–capsid interface. Again, these results suggest that the neutralizing effects of the drug are not immediately localized to the bottom of the canyon.

We proposed that the drug-binding cavity evolved to maintain flexibility of this region [23]. Empty cavities keep proteins at a higher energy state, where it will take less energy to cause structural changes. If the cavity itself plays a role in the viral life cycle, then it would explain why other picornaviruses (e.g., FMDV and mengovirus) have highly homologous structures in this region but do not have bound “pocket factors”. We proposed that the virus is binding to receptors in a two-step process [24]. The first step is a weak association with the south wall of the canyon. Subsequently, and in a temperature-dependent manner, conformational processes occur that increase the affinity of the virus for the receptor [25,26]. Capsid “breathing” [1] is likely an integral part of this receptor/capsid cooperativity that initiates the major conformational changes leading to the exposure of the myristylated N-termini so they can find their membrane target. As was noted previously [1], the locations of the digested termini are closer to the icosahedral 3-fold and 2-fold axes than the 5-fold axes. This has been supported by recent evidence that at higher temperatures, gaps are formed at the 2-fold icosahedral axes [27].

### 2.2. Antibody Neutralization of HRV and the Role of Capsid Flexibility

Antibodies are a major defense against picornavirus infection and several mechanisms of antibody-mediated neutralization have been proposed. Large changes in pI were observed when antibodies bound to virions [28,29,30,31]. This led to a popular hypothesis that antibody-mediated neutralization of picornaviruses is due to the induction of large conformational changes upon antibody binding. This contention was later supported by studies that suggested neutralization occurs with only 1–2 antibodies binding to the virion surface [32] that caused abrogation of viral attachment to the host cell [29,30,33] or prevent uncoating [34]. However, this result was challenged by subsequent monoclonal antibody studies on both HRV and poliovirus demonstrating that the neutralization numbers ranged from 6 to 20 antibodies and not 1–2 as previously suggested [35,36].

The hypothesis that neutralizing antibodies act by inducing conformational changes was directly tested by the determination of several antibody/virus complexes. Neutralizing monoclonal antibodies against HRV14 are divided into three groups: strong, intermediate and weak [35,36]. All strongly neutralizing antibodies bind to the Nim-IA site that is defined by natural escape mutations at residues D91 and E95 of VP1. Since strongly neutralizing antibodies form stable, monomeric antibody/virus complexes with a maximum stoichiometry of 30 antibodies per virion, it was concluded that they bind bivalently to the virions [35,36]. Weakly neutralizing antibodies form unstable, monomeric complexes with HRV14 and bind with a stoichiometry of ~60 antibodies per virion and strongly precipitate the virions. The remaining antibodies—all of which precipitate the virions—are classified as intermediate neutralizers [35,36].

#### 2.2.1. Structures of HRV14/Antibody Complexes

The structures of three different Fab/HRV14 (Fab17, Fab12 and Fab1) complexes and of one mAb/HRV14 (mAb17) complex have been determined [21,37,38,39,40]. Although all bind to the Nim-IA site, mAb17 and mAb12 are strongly neutralizing antibodies, whereas mAb1 is a weakly neutralizing antibody. The atomic structures of Fab1, Fab17 and HRV14 were used to construct pseudo-atomic models and then probed using site-directed mutagenesis [40]. In the Fab17/HRV14 complex, the loop of the Nim-IA site on HRV14 is clamped in the cleft between the heavy and light chain hypervariable regions and forms complementary electrostatic interactions with corresponding side chains of Fab17.

Fab17 and Fab12 both bind to the Nim-IA site at a somewhat tangential orientation that placed the constant domains (C_H1_C_L_) of 2-fold related Fabs in close proximity that could facilitate bidentate binding across icosahedral 2-fold axes. In contrast, Fab1 binds almost vertically to the virion surface with a “twist” that made it seem unlikely that these antibodies could bind bivalently. These studies, at resolutions of 20–30Å, consistently demonstrated that antibodies do not induce large conformational changes in the virion upon binding. In the case of the mAb17/HRV14 complex, it was thought that bivalent binding itself might induce conformational changes in the capsid. Bivalent binding was subsequently visualized in the structure of the mAb17/HRV14 complex [37]. Albeit at a limited resolution, this structure demonstrated that intact antibody, bound bivalently, also did not induced large conformational changes.

Since these cryo-EM studies were only a resolution of ~20Å, it is possible that smaller antibody-induced conformational changes went undetected. To this end, the crystal structure of the Fab17/HRV14 complex was determined [37]. In contrast to the prevailing dogma, the only observable changes in the virus were that the sidechains of VP1 D91 and E95 rotated slightly to form salt bridges with the basic residues in the paratope cleft. In fact, the major conformational changes were in the paratope region, where the CDR3 of Fab17 deformed to better fit the Nim-IA epitope. It was also apparent that Fab17 penetrates the receptor-binding canyon region. This might lead to the conclusion that Fab17 neutralizes by directly interfering with ICAM-1 binding. However, it has been shown that antibodies to all four antigenic sites can abrogate cell attachment [33] even though some sites (e.g., Nim-III) are distal to the canyon region. The simplest explanation for this is that, since the length of an antibody is the same as the viral radius, it would take only a few to interfere with virus–receptor interactions at the membrane surface. What is clear from these studies is that it is not at all necessary for highly efficacious antibodies to induce conformational changes in order to neutralize the virus.

#### 2.2.2. Antibodies to the Buried N-Termini Involved in Breathing

From the mass spectrometry results, HRV clearly undergoes a “breathing” process that exposes the N-termini of VP1 and VP4. Unlike the other regions of the capsid, these N-termini are highly conserved and, therefore, could be targets for a universal vaccine for HRV. To this end, monoclonal and polyclonal antibodies were raised against 30-residue peptides representing the N-termini of VP4 and VP1 [33]. Only the antibodies against the VP4 N terminus were found to neutralize viral infectivity in vitro. Because of the high degree of sequence conservation, the antiserum to HRV14 VP4 cross-reacted with serotypes HRV16 and HRV29. Antibody neutralization closely paralleled MALDI analysis of capsid “breathing” in that antibody neutralization and proteolysis were enhanced at 37 °C in the case of HRV16 but elevated temperatures were not required for either trypsin digestion or antibody neutralization with HRV14 and HRV29. Epitope mapping of the N-terminal 30 residues of VP4 suggested that the N-terminus of VP4 likely adopts a non-linear conformation in solution because antibody recognition of the peptides was sensitive to removal of 6–8 amino acids from either end. This was further substantiated by mutagenesis studies of the infectious HRV14 clone where serine at VP4 residue 5 was replaced with a cysteine. Surprisingly, all copies of VP4 of the S5C mutant formed cross-links but did not apparently affect virus viability. This suggests that the oligomer being formed could be a natural part of the breathing and infection process. While these studies suggest that VP4 might be useful as a pan-serotypic rhinovirus vaccine, the neutralization efficacy was far below what is observed with antibodies raised against the outer shell. This could be due to the oligomeric structure of the 30-residue peptide not appropriately mimicking that of the N-terminus being extruded from the capsid. However, it seems more likely that the weak reactivity is a function of epitope availability. The immune response is being forced to recognize an epitope that is only transiently being exposed whereas the NIm sites are always fully exposed in their natural conformation. As with other vaccine targets, there is a tradeoff between epitope conservation and exposure during B cell and antibody recognition.

#### 2.2.3. Do Antibodies Induce Conformational Changes in the Capsid?

A more recent study on a NIm-III monoclonal antibody (C5) yielded some interesting results on antibody recognition and capsid breathing [41]. C5 binds to the HRV14 NIm-III site that was defined by natural escape mutations in residues 72, 75, and 78 of VP3 [8]. This antibody was classified as an intermediate neutralizer and a weak aggregator [36]. For comparison, mAb17 and mAb12 described above are strong neutralizers that can decrease viral titers by more than five logs. The remaining viral infectivity is likely due to escape mutants rather than residual activity. Neither of these strongly neutralizing antibodies immunoprecipitated the virus under the conditions used for these assays. In contrast, C5 precipitates the virus to some degree and only decreases infectivity by less than three logs [36]. The weak precipitation suggested that C5 tended to bind bivalently to the capsid surface, but not as efficaciously as mAb12 or mAb14.

Fab fragments of C5 were added to HRV14 at 33 or 4 °C and the structures of the complexes were determined with cryo-EM. In all cases, Fab fragments were observed to bind to the capsid. The major difference was that there were more empty particles at 33 °C in the presence of the Fab than at 4 °C or in the absence of Fab at 33 °C. The treatment at 33 °C resulted in ~30%–60% of the particles releasing their genome, while only a small fraction of the virus was empty when treated at 4 °C. The major changes in the capsid upon release of the genome was expansion of the entire capsid and separation of the capsid at the icosahedral 2-fold axes. Interestingly, this separation of the VP2 α-helices at the icosahedral axes was discussed as a possible location of the N-termini extrusion in the previous mass spectrometry study [1]. There are relatively few intersubunit contacts here compared to the other icosahedral axes and it is immediately adjacent to the N-termini.

While it was suggested that the antibodies induced viral uncoating, it seems likely that the process is more complicated than that. From the studies reviewed above [22], the HRV capsid breathes at room temperature and this process is accentuated at higher temperatures. The hypothesis in this study was that the higher temperatures push the structural equilibrium of the capsid towards the “breathing” conformation and this structure is recognized by C5. This binding would then further push the structural equilibrium towards uncoating. If this hypothesis is correct, then C5 should prefer binding to the eclipsed particles rather than intact virions. This could be due to structural differences at the epitope between the two different particles or that the eclipsed particles might have more room for antibody binding than the native virions. However, the structures do not show evidence of either. C5 binds to both empty and full particles equally well since the apparent occupancy of C5 bound to both particles are the same when added at the same Fab/virus ratios. In addition, there is plenty of space for complete saturation of C5 on the capsid in both the native and eclipsed conformations. Perhaps more importantly, the structure of the NIm-III epitope region is essentially identical in the native particle and all four Fab/virus complexes presented in this work [27]. Therefore, C5 does not prefer binding to the eclipsed particle nor does there seem to be any significant structural changes around the epitope upon antibody binding.

These results are a clear demonstration of the changes in the HRV capsid upon release of the genome. However, it still is unclear if antibodies induce conformational changes upon binding and if this is a significant part of neutralization. Firstly, it is a bit of a misnomer to suggest that antibodies induce conformational changes per se. Even in this study, the model is suggesting that the higher temperatures are driving the changes in the intersubunit interactions and an antibody might be selecting for the altered conformation. However, even here, there is no obvious structural mechanism for either C5 recognition of the eclipsed particle or antibody induced conformational changes. In addition, if the main mechanism of C5 neutralization is the destabilization of the capsid, then one might expect some of the escape mutants would have stabilizing effects and lie distal to the epitope. Instead, all escape mutants to HRV14 monoclonal antibodies all lie on the capsid surface and are important antibody contact residues. Distal, stabilizing mutations are clearly possible as reviewed above in the case of escape mutations to the antiviral WIN compounds. Interestingly, distal antibody escape mutations are possible in viruses and discussed below in the review of mouse norovirus. Finally, it is unlikely that the common mechanism of antibody neutralization is induction of conformational changes. Indeed, all antibodies to the NIm-IA site in HRV14 have been shown to stabilize the capsid against pH denaturation rather than causing uncoating [35].

As previously reviewed [42,43], in any discussion about antibody-mediated neutralization of viruses, the development of the antibody response needs to be considered. The only driving force in B cell expansion is binding affinity of antigen to surface Ig of the B cell. There is no part of this expansion process that selects for neutralization efficacy, only binding. The only way for antibodies to be raised against the eclipsed particles is if there were significant amounts of this form of the virus during the adaptive immune response. Only then would it be possible for the antibodies to selectively bind to the eclipsed form and possibly shift the structural equilibrium. However, as was observed with the antibodies to the N-termini of VP4 described above, forcing an immune response to a form of the virus that is not the predominant species leads to weakly neutralizing antibodies. In this case, C5 is not as efficacious as the NIm-IA antibodies that were shown not to induce conformational changes [21,40] and, in fact, C5 binds to both native and eclipsed particles. The most likely scenario is that antibody binding to the dominant conformational species drives the selection and production of antibodies. These antibodies can theoretically aggregate, stabilize, or bind to eclipsed particles [36,43]. However, as previously discussed [42], it seems most likely that the in vivo synergism of antibodies with other components of the immune response plays the dominant role in antibody-mediated protection in vivo.

## 3. Mouse Norovirus (MNV)

Noroviruses are the major cause of epidemic gastroenteritis in humans and, as such, are important pathogens (for review, see [44]), causing ~20 million cases annually, resulting in more than 70,000 hospitalizations and 570–800 deaths in the US alone. While not often a fatal disease in the developed world, norovirus infections are estimated to cost more than $2 billion per year for health care and lost productivity. Controlling the spread of norovirus is challenging, since as few as ten virions are sufficient to infect an adult [45].

Efforts to make effective norovirus vaccines have been hampered by our lack of understanding of the structural mechanisms of viral escape. In addition, noroviruses are constantly evolving and generate new strains every 2–4 years [46,47,48] that result in worldwide epidemics [48,49]. Developing efficacious vaccines requires a detailed understanding of how escape mutations block antibody binding and the limitations in altering the virus capsid to evade the immune system. Such studies have been difficult with human noroviruses. While there have been advances in cell culture methods [50,51], the lack of small animal models have made in vivo analyses more difficult [52].

**Figure 4:**
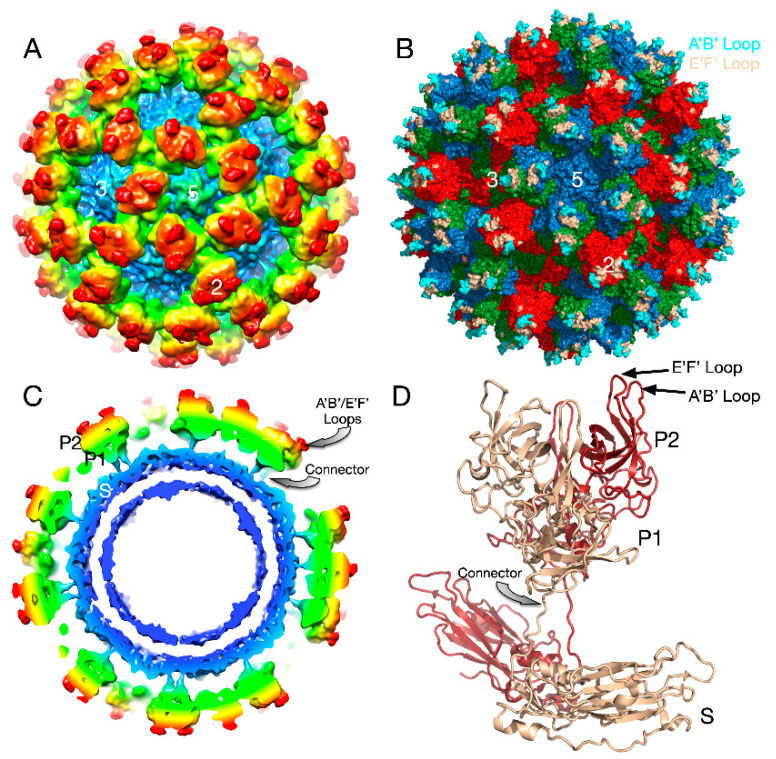
Structure of apo MNV. (**A**) The 8Å cryo-EM structure of MNV colored according to radial distance. (**B**) Pseudo-atomic model using the atomic model of the P domain [61] and the shell domain [2]. A, B, C subunits are colored blue, green, and red, respectively. The A’B’ and E’F’ loops are colored cyan and tan, respectively. (**C**) Cross-section of the model from (A) showing the P domain “floating” above the shell domain by more than 18Å. (**D**) The pseudo-atomic model using the 8Å image reconstruction and the atomic models of the S and P domains. Dimer subunits are colored red and tan, respectively.

Caliciviruses are T = 3 icosahedral particles with 180 copies of the major capsid protein (VP1; ~58 kDa), that is divided into the N-terminus (N), the shell (S) and C-terminal protruding (P) domains [53,54,55,56]. The S domain forms a shell around the viral RNA genome, while the P domains dimerize to form protrusions on the capsid surface. The P domain is subdivided into P1 and P2 subdomains, with the latter containing the binding sites for cellular receptors [57,58] and neutralizing antibodies [59,60,61]. The overall architecture of mouse norovirus is shown in Figure 4, with the three copies of VP1 in the icosahedral asymmetric unit being designated as subunits A (blue), B (green), and C (red). Also noted in this figure is the location of the A’B’ and E’F’ loops in the P2 domain that will be discussed in detail below.

Calicivirus capsid flexibility likely plays important roles in receptor binding and escape from immune surveillance. There are at least two aspects of capsid flexibility; the entire P domain freely moves about the capsid surface and the conformation of the P domain itself is highly flexible and sensitive to antibody escape mutations and receptor binding.

### 3.1. The First Mode of Flexibility; “Floating” P Domains

The cryo-EM structure of MNV was determined to a cryo-EM resolution of ~8Å [56,61]. The obvious difference between this structure and the crystal structure of human Norwalk virus (NV) [56,61] was that the P domains of NV VLPs rest upon the shell domain, while there is a large gap in the electron density between the shell and protruding domains of MNV-1 (Figure 4). This gives the appearance that the MNV-1 P domains lift off the shell (S domains) to form a second proteinacous layer. As shown in this figure, there is a linker region between the S and P domains that is coiled up in NV and becomes extended in MNV.

Since this unusual “floating P domain” conformation (“extended state”) was so different than the crystal structure of NV (“contracted state”), the structure of rabbit hemorrhagic disease virus (RHDV) VLP was also determined [61]. Similar to MNV-1, the P domains are lifted off the surface of the shell and have diffuse density, suggesting a high degree of flexibility. Neither MNV (genotype GV.1) nor RHDV (genotype GIV) infect humans and it is possible that it is a feature not found in the human noroviruses. Therefore, the cryo-EM structure of the human Vietnam026 (GII.10) VLP was determined [62]. The cryo-EM reconstruction of the GII.10 norovirus VLP at ~10 Å resolution showed features very similar to MNV and RHDV. As with MNV and RHDV, the P domain appeared as a second outer shell with the P domain raised off the S domain by ~15 Å. Importantly, these studies also showed that this apparent P domain flexibility may play an important role in antibody binding. Hansman et al. determined the crystal structure of the P domain complexed with the Fab fragment from an antibody (5B18) that broadly recognized different GII viruses [62]. The crystal structure of the P domain/Fab complex showed that the 5B18 Fab bound to a conserved region of the protruding domain. This binding site is involved in interactions with other regions of the capsid and is partially buried in the virus particle in the “compressed” state. Despite the occluded nature of the recognized epitope in the VLP structure, ELISA binding indicated that the 5B18 antibody was able to capture intact VLPs. The base of the P domain is only exposed to the antibody if there is extreme flexibility in the tether region between the shell and the P domain. This result has been further substantiated by more recent results in genotypes GI.1 [63] and GII.4 [64], where various epitopes are clearly buried in the compressed particle and only exposed if the P domain is allowed to lift off the shell.

### 3.2. MNV–Receptor Interactions

The flexibility of the entire P domain may also play a role in receptor interactions. The Virgin lab found that the cell receptor for MNV was proteinaceous and was not dependent upon carbohydrates for binding [65]. Using the CRISPR-Cas9 system, a gene that encodes a cell-surface protein that contained an immunoglobin domain and belonged to a lipid protein family was most significantly enriched in the surviving cells, CD300lf. It was confirmed that this was the receptor from studies showing that knocking out Cd300lf in BV2 cells blocked infection by MNV and treating the cells with antibodies to CD300lf blocked MNV attachment. Attachment of MNV to BV2 cells was not affected by pre-treatment of the cells with the mannosidase I inhibitor, kifunensine, suggesting that carbohydrates do not play a significant role in MNV attachment. Even stronger evidence of the importance of CD300lf came from the demonstration that expression of this mouse cell surface protein in HeLa cells made these human cells susceptible to MNV infection. While carbohydrates are not apparently important for cell binding, they found that bile salts such as glycochenodeoxycholic acid (GCDCA) enhances viral attachment to BV2 cells [66]. This is clearly a specific interaction since a chemically similar salt, taurocholic acid (TCA), had no effect on binding. These results were further substantiated using isothermal titration calorimetry that showed GCDCA binds to the expressed form of the P domain with Kd of ~6 µM, but TCA did not bind at all.

Using these soluble forms of CD300lf and the P domains, they also directly measured the interaction affinity using surface plasmon resonance [66]. The monomeric CD300lf protein bound to the P domain with a Kd of ~219 µM. While this represents a very weak interaction, the other members of the CD300 family, that failed to confer susceptibility to MNV infection (mouse CD300ld, mouse CD300lh, and human CD300f), showed little to no binding. By adding calcium, the affinity improved to ~25 µM and when GCDCA and calcium were both added, the affinity improved to ~12 µM. The monomeric CD300lf molecules were then linked as dimers by fusing them to an antibody Fc fragment. As expected, this improved apparent affinity (avidity) to ~0.5 µM. This is entirely consistent with foundational studies demonstrating that Fab fragments bind with ~100–1000 lower apparent affinity than the corresponding IgG [67]. The neutralizing antibody, A6.2, was able to block binding of this bivalent CD300lf molecule, suggesting that at least in vitro antibody neutralization is due to blocking of receptor attachment.

**Figure 5:**
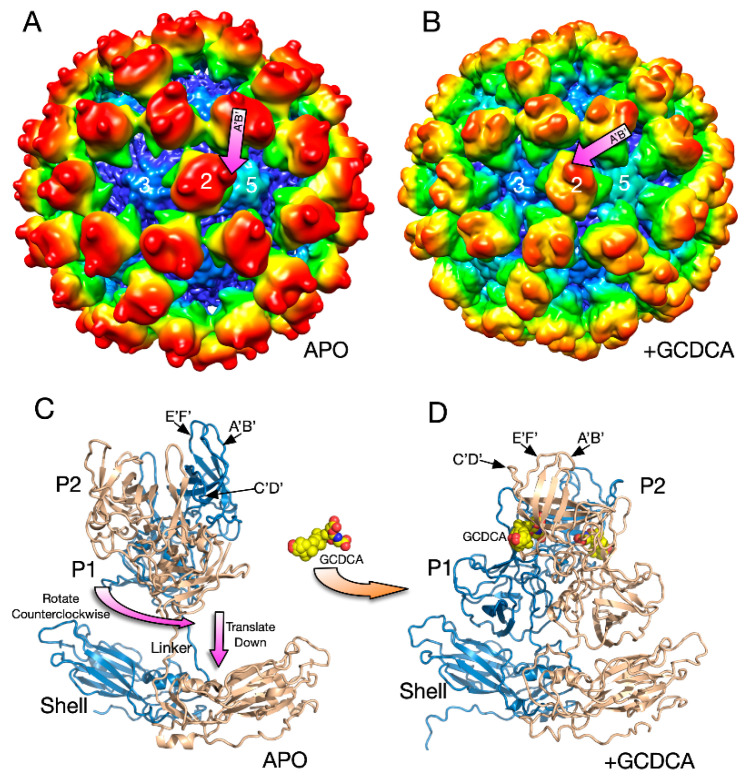
Structural changes in the MNV capsid upon the addition of the bile salt, GCDCA. (**A**) The 8Å structure of apo MNV and (**B**) the 3Å structure of MNV in the presence of GCDCA and low pass filtered to 8Å for comparison with the apo structure. A and B are colored from blue to red according to radius. (**C**) The pseudo-atomic model of the apo structure of MNV using the 8Å and 3Å cryo-EM electron density maps. (**D**) When GCDCA is added, the P domains rotate by ~90° and rest on top of the shell.

To determine the atomic details of CD300lf binding to the receptor, the atomic structure of the soluble P domain/CD300lf complex was determined [66,68] followed by the cryo-EM structure of the whole virus/CD300lf complex [2]. Initial attempts at determining the cyo-EM structure of the whole MNV virus/CD300lf complex failed to clearly show receptor binding to the P domain. This could have been due to the relatively low affinity of CD300lf in the absence of GCDCA and/or the marked flexibility of the P domain causing disorder in the reconstruction. Therefore, as a control, the cryo-EM structure of the MNV/GCDCA complex was determined. Surprisingly, the addition of GCDCA alone cause large conformational changes in the capsid (Figure 5).

There were several major changes in the MNV capsid upon the addition of GCDCA [2]. The P domains rotated by ~90° counterclockwise and drop down onto the surface of the shell domain (Figure 5). Likely because of the new and extensive interactions between the shell and P domains, the P domain became highly ordered and the entire capsid protein could be traced. The structure of the P domain was essentially identical to the crystal structure of the GCDCA/P domain complex [66,68]. GCDCA was observed to bind between the P domain dimers and the C’D’ loop flips up towards the A’B’ and E’F’ loops. This, in turn, pushes the E’F’ loop towards the A’B’ loop causing the P domain to have the “closed” conformation observed in the original crystal structure [61]. While GCDCA was clearly observed between the two P domains in the cryo-EM density, there was no evidence of it binding anywhere else in the structure that might cause this large P domain motion. It was suggested that the effects of GCDCA might be non-specific via changes in the properties of water around the P domain [2].

**Figure 6:**
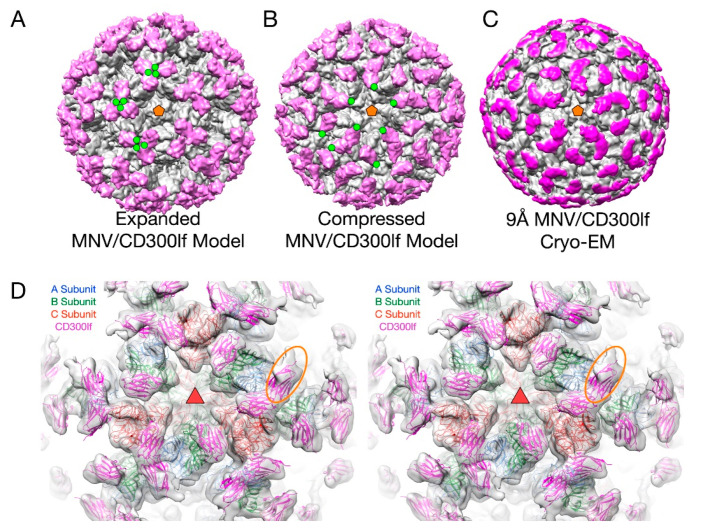
The structure of MNV complexed with the receptor, CD300lf. In panels A–C, the virus is grey and the receptor mauve. (**A**) The model for the expanded form of MNV with the CD300lf place using the atomic structure of the P domain/CD300lf complex [66]. An icosahedral 5-fold axis is represented by an orange pentagon and the C-termini of some of the CD300lf molecules are highlighted with green circles. (**B**) An atomic model of the MNV/GCDCA/CD300lf complex using the atomic model of the Pdomain/CD300lf and the EM structure of the MNV/GCDCA complex. (**C**) The 9Å cryo-EM structure of the MNV/GCDCA/CD300lf complex [2]. (**D**) The model from panel B overlaid with the cryo-EM density of the MNV/GCDCA/CD300lf shown in panel C. An icosahedral 3-fold axis that is denoted by the orange triangle. The orange ellipse notes the CD300lf bound to the A subunit with weaker density likely due to lower occupancy.

This flexibility of the P domain may play an important role in receptor interaction in MNV by creating more space for the receptor to bind [2,66]. In Figure 6, the atomic structure of the MNV P domain/CD300lf receptor [68] was modeled into the pseudo-atomic cryo-EM structures of MNV in the apo (Figure 6A) and the 3Å MNV/GCDCA complex structure (Figure 6B). For comparison, the ~9Å cryo-EM structure of the MNV/GCDCA/CD300lf complex is shown in Figure 6C. The overlay of the complex structure in the cryo-EM density is shown in Figure 6D. A, B, and C subunits are colored blue, green, and red, respectively and the CD300lf receptor is shown in yellow. In all panels, the C-termini of the CD300lf N-terminal fragment is noted as green circles on some of the copies of the receptor on the viral surface. As shown in the model of the expanded form, the C-termini of the CD300lf form trimers in the icosahedron. There is clearly not enough room for all the P domains to be saturated with receptor. When the structure of the GCDCA bound “contracted” structure [2] of MNV is used for modeling, there is additional space around each of the P domains for at least a higher degree of saturation with receptor. Interestingly, the P domains of the A subunits around the 5-fold axes are oriented such that there is clearly room for saturation. While this figure shows just two conformations, it is more than likely that the flexibility of the linker allows for multiple conformational states. These results suggest that when MNV enters the gut with its high levels of bile salts and other solutes, the virus may switch from the extended to the contracted state to make more space available for receptor binding.

The P domain mobility could also affect antibody recognition. In the expanded state, the flexible tether between the shell and the P domains could be sensitive to proteases. This could release soluble P domains to act as a “smoke screen” for the immune system and present antigenic sites not normally exposed on the viral surface. Similarly, the marked flexibility of the P domains in the expanded state might expose these antigenic sites without having to be cleaved from the shell. In either case, if any of these normally buried sites are immunodominant, then the immune response might focus on producing antibodies that bind poorly or not at all to the contracted capsid. This hypothesis is supported by the findings that the immune response to MNV included antibodies to the buried shell domain [69] and from studies on human genotypes GII.10 [62], Gi.1 [63], and GII.4 [64]. Essentially, the highly flexible nature of the P domain could be a “moving target” for the immune response.

### 3.3. The Second Mode of Flexibility; within the P Domains

From the results above, the P domain is like a balloon floating above the viral shell, attached only by a thin tether. The P domain is structurally isolated from the rest of the capsid and, therefore, it seems highly unlikely that antibodies or receptors can bind to the P domain and transmit conformational changes to the shell. However, there is growing evidence that the P domain itself is highly flexible and that this motility is necessary for viral function and antibody escape.

### 3.4. MNV P Domain Flexibility and Antibody Escape

In the initial crystal structure of the MNV P domain [61], the outer two loops (A’B’ and E’F’) displayed two discreet conformations—a closed structure where the two loops are tightly associated and an open structure where the loops are splayed apart.

To understand how noroviruses escape antibody neutralization, we determined the cryo-EM structure of MNV complexed with Fab fragments from a neutralizing A6.2 [56]. It was clear that A6.2 bound to the outermost tip of the P2 domain, right where the A’B’ and E’F’ loops lie. To better understand the interaction of mAb A6.2 with the P domain, we determined the crystal structure of the A6.2 Fab to ~2.5 Å [70]. Typically, the third hypervariable loop (CDR3) of the heavy chain makes most of the contact with the epitope. Interestingly, this loop in A6.2 is strongly hydrophobic with the sequence “YFYALDYW”. When the structures of A6.2 and the P domain dimer were placed into the cryo-EM density, it was evident that A6.2 fit better onto the open conformation than the closed, with the hydrophobic CDR3 loop extending into the hydrophobic cleft between the A’B’ and E’F’ loops. Those hydrophobic residues in the P domain are deeply buried under the tips of the A’B’ and E’F’ loops in the closed conformation and not accessible to mAb A6.2. In addition, the CDR3 loop of A6.2 completely overlaps the E’F’ loops when modeled into the closed conformation, making it impossible for A6.2 to bind. Taken together, the modeling strongly suggested that A6.2 prefers the open conformation both in terms of structural and hydrophobic complementarity.

Using the A6.2/MNV-1 docking model [56,61] as a guide for mutagenesis, we identified six single point mutations in the E’F’ loop of the MNV-1 P domain that completely abrogated mAb A6.2 binding to MNV-1 and allowed mAb A6.2 neutralization escape in culture and in mice [70]. These studies suggested that a number of escape mutants block antibody binding by limiting the conformational repertoire of the E’F’ loop and not via direct interactions with the antibody. These studies are the first to suggest that escape mutations may act by limiting flexibility of an epitope or by driving the conformation towards a structure not recognized by the antibody. For example, in the open conformation, the L386F mutation would place the larger Phe side chain directly in contact with the bound antibody and would be exposed to water. However, in the closed conformation, the PHE would be completely buried between the A’B’ and E’F’ loops. Hence, the L386F mutation would appear to push the structural equilibrium towards the closed conformation, to which A6.2 cannot bind. Similarly, the A382R mutation in the closed conformation places the Arg into the solvent and thus should be the favorable structure. In contrast, in the open conformation, the A382R mutation would place the Arg side chain into a cluster of basic residues. Again, this suggests that the plastic P domain can thwart antibody binding by shifting the structural equilibrium away from the conformation favored by the neutralizing antibody.

**Figure 7:**
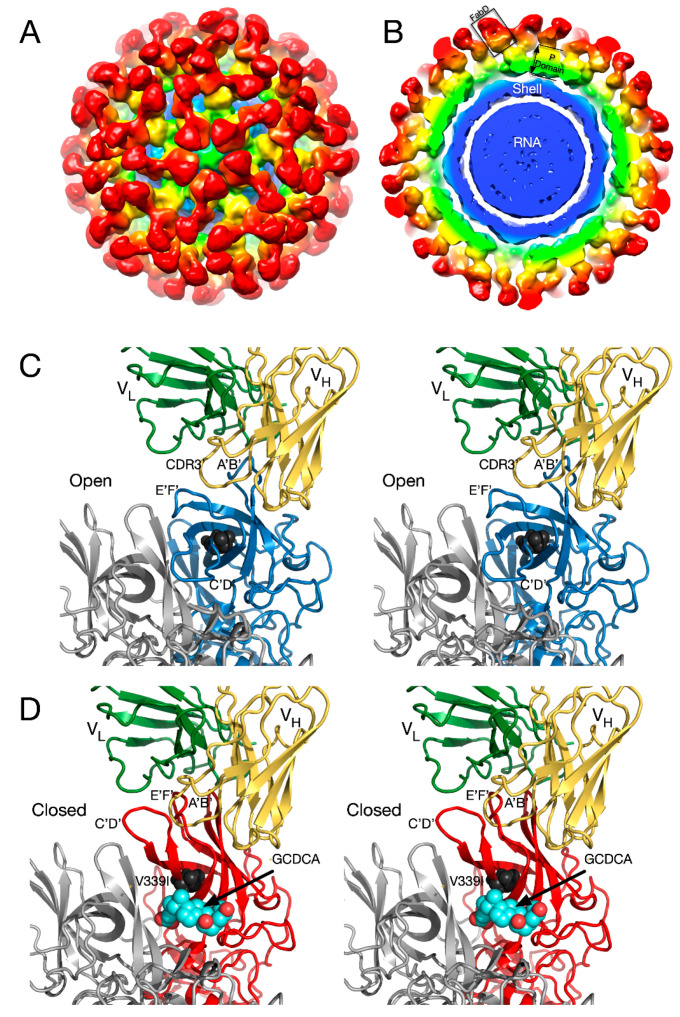
Structure of the Fab 2D3.7/MNV complex and possible effects of bile salts on antibody binding. (**A**) ~9.0Å cryo-EM density of the FabD/MNV complex viewed down an icosahedral 5-fold axis. (**B**) Central section of the FabD/MNV electron density map. The approximate colors of the shell, P1, and P2 domains are blue, green, and yellow, respectively. The FabD variable domains are orange and the constant domains, red. (**B**) FabD modeled into the cryo-EM density using the “open” conformation of the P domain. The location of the “allosteric-like” escape to this antibody (V339I) is shown in black. (**C**) Stereo diagram of the FabD bound to the “open” conformation. **(D)** Stereo diagram of the hypothetical model of FabD bound to the “closed” conformation induced by the binding of bile salts to illustrate the antibody/P domain clashes in this conformation.

An even more extreme case of P domain plasticity was observed with a second neutralizing antibody, 2D3 (Figure 7). Compared to A6.2, MNV1 had far more difficulty in overcoming neutralization to 2D3, as it took over 20 passages for MNV1 to escape mAb 2D3 neutralization [69,70]. The escape mutants to A6.2 were still neutralized by 2D3 and visa-versa. Importantly, all the MNV strains tested were neutralized to at least some degree by 2D3 while A6.2 was far more selective. This suggested that the epitope on the P domain region recognized by 2D3 was significantly different and more conserved than that for A6.2 and could, therefore, make a good vaccine target.

To understand the difference between these two antibodies, the cryo-EM structure of the 2D3 bound to MNV was determined [69] (Figure 7). Like A6.2, 2D3 appears to bind to the open but not the closed conformation of the P domain. While 2D3 contacts the E’F’ loop of the P domain similar to mAb A6.2, mAb 2D3 appears to bind slightly deeper in the crevice between the A’B’ and the E’F’ loops. Surprisingly, neither of the natural escape mutants to 2D3 (D348E and V339I) are in contact with the bound antibody. Just as odd is the fact that even though some of the escape mutations to A6.2 are in contact with 2D3, none of them affect 2D3 neutralization. What is so special about the 2D3 contact area that severely restricts the repertoire of possible mutations so that only “allosteric” escape mutants were isolated?

To understand how the 2D3 escape mutants, that are distal to antibody contact and buried beneath the surface, can block 2D3 binding, dynamic simulations were performed on the P domain where the V339I mutation was modeled into both the open and closed conformations [71]. The results for the three simulations indicated that FabD/MNV-bound complex is sensitive to the flexibility of P domain dimer interfacial interactions, notably the salt bridge network that undergoes rearrangements necessary to accommodate the antibody binding. The buried dimer interface salt bridge network is complex and dynamic allowing the dimer to adopt more than one conformation. The V339I escape mutation is not in either of the A’B’ or E’F’ binding loops and simulations show it changes the salt bridge network and restricts the molecular liberation of the interfacial region and thus the conformational flexibility.

Together, these results strongly suggest that the P domain is in a dynamic structural equilibrium where the crystallographically observed open and closed states represent two possibilities of a montage of conformations. The site directed escape mutations to A6.2 and the naturally occurring escape mutations to 2D3 suggest that the virus can block antibody binding by shifting this equilibrium towards the closed state. If true, then it necessarily follows that the closed state is the viable and infectious form of the virus. However, it is not at all clear why the 2D3 contacts on the P domain are so immutable and suggests that this region has an important role.

### 3.5. MNV–Receptor Interactions

The details of the MNV–receptor interactions have been elucidated with the atomic structure of the P domain/CD300lf complex [66,68] and the cryo-EM structure of the MNV/CD300lf complex [2]. The CD300lf contact site is near the top of the P2 domain, between the A’B’ and D’E’ loops. Interestingly, this contact surface includes some of the amino acids involved in the binding of the neutralizing antibody, A6.2. From structural analysis, they also found that that that GCDCA and lithocholic acid (LCA) bind in two deep pockets in the P domain dimer interface, distant from receptor and antibody binding sites [66].

The conformation of the A’B’ and E’F’ loops in the receptor complex is nearly identical to that of the closed conformation. The major difference is that the C’D’ loop is “turned up” compared to the P domain alone. This affords enough space for the bile salt, GCDCA (tan spheres), to bind. It may be that the structural equilibrium in solution is shifted by GCDCA binding to the closed form, which may be preferred by CD300lf. In this way, the bile salts may enhance receptor binding, indirectly, by altering the dynamics and structural equilibrium of the P domain. What is particularly interesting is that the V339I escape mutant (black spheres) lies immediately adjacent to the bile salt binding site. As with the bile salts, we had proposed that the V339I mutation shifted the equilibrium towards the closed conformation that does not favor antibody binding [61,70,71]. It is rather exciting to consider that the V339I escape mutant and bile salts drive the P domain conformation towards the closed conformation that favors receptor binding and at the same time away from the open conformation favored by antibody binding.

The conformation of the P domain in solution is more than likely a montage of structures including the observed open and closed configurations. This structural equilibrium can be disrupted by environmental cues and antibody escape mutants. In the open conformation, the A’B’ and E’F’ loops are splayed apart, making room for antibodies (e.g., A6.2 and 2D3) to bind. The closed conformation does not expose the hydrophobic region between the two loops and the E’F’ loop clashes with the CDR3 loop, therefore, preventing antibody binding. All of the escape mutations to 2D3 are distal to the antibody binding contact and dynamic simulation studies with the V339I escape mutation suggest that it causes long-range structural disruption in the P domain dimer and may be pushing the structure from the open (that binds antibody) to the closed (that does not bind antibody) conformation [71]. Similar to the V339I escape mutant, compounds in the gut milieu such as calcium and bile salts may also push the structural equilibrium towards the closed conformation to which the receptor binds [66]. Interestingly, the location of V339I is immediately adjacent to the bile salt-binding site in MNV and may be mimicking the effects of bile salts. While clearly this simplistic model needs to be extensively tested, it suggests that the P domain in solution is sampling many conformations waiting for the right environmental cues when it is in the best location for infection. It is fascinating that apparently the predominant structure presented to, or recognized by, the immune system is the open conformation, while the conformation necessary for receptor binding is closed. This could be akin to the “camouflage” model proposed to FMDV, where the integrin-binding RGD motif is on a highly mobile loop that presents many different conformations to the immune system [72,73]. It is important to note that P domain flexibility is likely to play an important role in all of the caliciviruses as suggested in the case of human noroviruses interacting with their carbohydrate receptor (for a review, see [74]).

## 4. Summary

From these two cases, viruses with simple protein shells are more dynamic and flexible than might be expected. In the case of the rhinoviruses, the capsids appear to be breathing where the capsid may be opening and closing around the 2-fold axes and exposing the myristoylated N-termini of VP4. The receptor, ICAM, may prefer one of the conformations sampled during this breathing process and begin the uncoating process. As suggested by the effects of the WIN compounds and mutagenesis studies, the empty drug-binding pocket minimizes the energy required for these conformational changes. While it is attractive to suggest that antibody binding might similarly drive the capsid towards uncoating, the fact is that the most potent antibodies do not cause conformational changes. Even in the one study reviewed here that suggests such conformational changes might occur, the antibody does not appear to prefer one conformation over the other. In the end, the immune system simply recognizes epitopes presented to it and does not select for antibodies that induce conformational changes. While it is attractive to consider vaccines that recognize viruses in a transition state, trying to force the B cells to recognize a conformation that is not prevalent in solution will likely yield a poor adaptive immune response.

While the rhinoviruses appear to breathe in preparation for receptor interactions and uncoating, MNV has several modes of flexibility for quite different reasons. MNV has a highly mobile P domain that can move around the capsid surface that likely facilitates binding to the target cell. This flexibility of the P domain might also be involved in avoiding the immune system by presenting antigenic sites exposed in one state but not the other. This gross movement of the P domain is clearly sensitive to environmental conditions and appears to improve virus binding to the receptor. Similarly, there is evidence that the structure of the P domain itself responds to environmental cues. In physiological phosphate-buffered saline, the C’D’, E’F’, and A’B’ loops adopt an “open” conformation that appears to be recognized by antibodies. As the virus enters the gut, solutes such as bile salts move these loops to the “closed” conformation that is no longer recognized by antibodies but is favored by receptors. So, unlike HRV14, the plastic nature of the capsid allows the virus to present one “face” to the immune system and a very different one for cell attachment. Nevertheless, it is clear that neither of these viruses are simply inanimate “tin cans”.
